# Tobacco smoking and alcohol drinking among HIV infected people using antiretroviral therapy

**DOI:** 10.18332/tid/86716

**Published:** 2018-04-24

**Authors:** Dharma N. Bhatta, Arjun Subedi, Narbada Sharma

**Affiliations:** 1 Department of Community Medicine and Public Health, People’s Dental College, Tribhuvan University, Kathmandu, Nepal; 2 Center for Tobacco Control Research and Education, Helen Diller Family Comprehensive Cancer Center, University of California, San Francisco, United States; 3 Program Planning Department, Society for Local Integrated Development (SOLID Nepal), Kathmandu, Nepal; 4 HIV/AIDS Treatment, Prevention and Control Center, Sukraraj Tropical and Infectious Disease Hospital, Ministry of Health and Population, Kathmandu, Nepal

**Keywords:** sex worker, ART, alcohol, tobacco

## Abstract

**INTRODUCTION:**

Tobacco smoking, common in people living with human immunodeficiency virus (HIV), is associated with increased mortality and morbidity. This study aimed to determine the proportion of current smokers, as well as assess the factors associated with tobacco smoking and drinking alcohol, among people living with HIV (PLHIV) in Nepal.

**METHODS:**

A cross-sectional study was conducted at an antiretroviral therapy (ART) clinic in Kathmandu, Nepal between September and December 2014. Data were collected among 132 HIV infected individuals using a random sampling technique and face-to-face interview. Binary logistic regression analysis was performed to estimate the factors associated with current tobacco smoking and drinking of alcohol.

**RESULTS:**

Among the HIV infected people, the proportion of current tobacco smoking was 26.5% (95% Confidence Interval (CI): 18.9-34.1), while drinking of alcohol was 22.7% (95% CI: 15.5-30.0). The respondents who were infected with HIV, after sexual contact with sex workers, were more likely to smoke tobacco (OR=15.2, 95% CI: 4.35-53.08) and drink alcohol (OR=4.50, 95% CI: 1.70-11.93) than those who were infected from drug needle use and blood transfusion. HIV infected individuals, who forgot to take ART medication, were three times more likely (OR=3.17, 95% CI: 1.36-7.38) to drink alcohol than those who did not forget to take ART medication.

**CONCLUSIONS:**

Proportion of people who smoke tobacco and drink alcohol is high among the HIV infected individuals who had sexual contact with sex workers in Nepal. There is an urgent need to develop immediate, sustainable and efficient programs to control tobacco smoking and alcohol drinking among vulnerable populations in low and middle-income countries like Nepal.

**ABBREVIATIONS:**

HIV: Human Immunodeficiency Virus, AIDS: Acquired Immunodeficiency Syndrome, ART: Antiretroviral Therapy, PLHIV: People Living with Human Immunodeficiency Virus, CI: Confidence Interval, STIDH: Sukraraj Tropical and Infectious Disease Hospital, OR: Odds Ratio, SD: Standard Deviation

## INTRODUCTION

Tobacco smoking is very common among HIV infected people, estimated at approximately 50 to 70 percent^1^. In Nepal, 47% of HIV infected people were current tobacco smokers^2^. Mortality and morbidity were reduced and life expectancy increased among HIV infected people after the introduction of antiretroviral therapy (ART)^3^. In addition, non-AIDS related cardiovascular disease and malignancies were reported to be the main causes of death amongst people living with HIV (PLHIV) compared to the general population^4-6^. However, death caused by the side effects and the long-term toxicity of ART amongst PLHIV remain unclear^7^. Smoking tobacco among PLHIV alters immune and virological responses, leading to increased vulnerability to infection, tuberculosis and low adherence of ART^8,9^. On the other hand, life expectancy was found lower among tobacco smoking PLHIV than in non-smoking PLHIV^10^. Tobacco products were associated with higher mortality among PLHIV compared with just being infected with HIV^11^. Unemployment, alcohol use, illicit drugs, low ART adherence, low social support, low education level, decreased CD4 cells count and discriminations were found significantly associated with tobacco smoking amongst PLHIV^12^.

Tobacco smoking increased among PLHIV with alcohol consumption^13^. Drinking of alcohol was higher among PLHIV than the general population^14^. Alcohol consumption accounted for more deaths among PLHIV than by only HIV and other diseases^15^. Consumption of alcohol produced a toxicity that reacted with ART, resulting in elevated risk of liver diseases, co-infections, development of other diseases, as well as absorption and metabolism of drug and immune dysfunction^16^. Non-adherence to ART, decline of CD4 cells count, increased viral load, rapid development of diseases and delayed on care of HIV were the factors associated with drinking alcohol^17,18^. In addition, risky sexual behavior was also linked to drinking alcohol, which increased the risk of HIV transmission^19^.

The HIV epidemic is high in developing countries. Tobacco and alcohol related health risks are more common among PLHIV. Very few studies are available in Nepal related to the problems of PLHIV. Thus, we aimed to determine the proportion of people who smoke tobacco and drink alcohol, as well as assess the factors associated with tobacco smoking and drinking of alcohol, amongst PLHIV in Nepal.

## METHODS

### Study design, settings and participants

A cross-sectional study was conducted among 132 HIV infected individuals who received antiretroviral therapy (ART) at the ART clinic of the Sukraraj Tropical and Infectious Disease Hospital (STIDH) – a national-level government health care facility for infectious disease control in Kathmandu, Nepal. The STIDH is one of the largest ART clinics in Nepal that provides HIV counseling, testing, nutritional supports, antiretroviral medicines, medical care, opportunistic infection treatment and medicine services. HIV-infected individuals aged 18 years and older were eligible participants of the study. All the participants were randomly selected. We developed a list of 1447 ART receiving clients. The first list was developed using a simple random sampling technique that included 132 clients; the second list was developed when we were not able to interview the clients from the first list. We calculated sample size using a conservative formula with 50% prevalence, 95% confidence level, 10% margin of error and 30% no-response, resulting in a total sample estimation of 132 HIV infected individuals. The study was conducted between September and December 2014 and the response rate was 100%.

A face-to-face interview was conducted with individual participants in a separate room of the ART clinic by the team of trained interviewers using a structured questionnaire. Information was collected with the pre-tested questionnaire in the Nepali language. All the participants were informed about the study objectives and procedures. A written informed consent was obtained from each participant before interview. Firstly, ART numbers were used during random selection of the sampled participants, and later separate numeral codes were developed to ensure confidentiality. This study was approved by institutional ethical review committee of Sukraraj Tropical and Infectious Disease Hospital (STIDH), Kathmandu, Nepal.

### Measures

Measures incorporated in the study were demographic and health variables, ART adherence, tobacco smoking and drinking of alcohol. Demographic information obtained from the HIV infected individuals were age, gender, ethnicity, religion, occupation, education, marital status, income, and number of children; health related information included age at HIV diagnosis, duration of ART received, mode of HIV infection, HIV status of the spouse, clinical stage, known co-morbidities, and most recent CD4+ T cell count.

The ART adherence was assessed using the question: ‘have you ever forgotten your HIV medication?’; with answer ‘yes’ or ‘no’.

### Outcome variables

Tobacco smoking was assessed using the question: ‘are you a current tobacco smoker?’; with answer ‘yes’ or ‘no’. Similarly, alcohol drinking was assessed using the question: ‘are you a current alcohol user?’; with answer ‘yes’ or ‘no’.

Contents of the study instruments were validated with two independent experts and were pre-tested among fifteen HIV infected people. Pre-tested data were only used to refine the questionnaire and were not included in the final analysis.

### Data analysis

EpiData version 3.1 was used for data entry. The completeness and the accuracy of the data were checked before and after data entry. The R-software was used to estimate the proportion and other outcomes of the study variables of interest. Significant differences between demographic, health variables and substance use (tobacco and alcohol) were performed by using Fisher’s exact test. Binary logistic regression analysis was performed to determine the factors associated with tobacco smoking and alcohol drinking. The unadjusted odds ratio was presented with 95% confidence interval. A p<0.05 was considered as significant.

## RESULTS

The mean (SD) age of the participants was 36.1 (7.8) years. The majority (53.0%, 95% CI: 44.4-61.6) of the participants were female. Most of the participants followed the Hindu religion (70.5%, 95% CI: 61.9-78.1) and were of non-indigenous ethnicity (56.1%, 95% CI: 47.5-64.6) (Table 1). One-third (36.4%, 95% CI: 28.2-45.2) of the participants had not attained formal education. Most of the participants (84.8%, 95% CI: 78.6-91.0) had children and among them 79.5% (95% CI: 70.8-86.5) had ≤ 2 children. Monthly per capita income was low, with 64.4% (95% CI: 55.6-72.5) of the participants earning≤ 5000 NPR (1USD=100NPR) (table not shown).

**Table 1:** Demographic, behavioral and health characteristics of HIV infected people (N=132)

*Characteristics*	*n (%)*	*95% CI*
**Age in years [mean (SD): 36.1 (7.8)]**		
≤ 36	77 (58.3)	49.4-˗66.8
>36	55 (41.7)	33.1-˗50.6
**Gender**		
Male	62 (47.0)	38.3-˗55.6
Female	70 (53.0)	44.4˗-61.6
**Ethnicity**		
Non-indigenous	74 (56.1)	47.5-˗64.6
Indigenous	58 (43.9)	35.4-˗52.5
**Religion**		
Hindu	93 (70.5)	61.9-˗78.1
Others	39 (29.5)	21.9-˗38.1
**Occupation**		
Unemployed	43 (32.6)	24.7-˗41.3
Informal employee	48 (36.4)	28.2˗-45.2
Formal employee	41 (31.1)	23.3-˗39.7
**Current tobacco smoking (last 30 days)**		
Yes	35 (26.5)	18.9-˗34.1
No	97 (73.5)	65.8-˗81.1
**Current alcohol drinking (last 30 days)**		
Yes	30 (22.7)	15.5-˗30.0
No	102 (77.3)	70.0˗-84.5
**Age at HIV diagnosed [mean (SD):34.1(7.9)]**		
≤ 34 Years	75 (56.8)	47.9-˗65.4
> 34 Years	57 (43.2)	34.6-˗52.1
**Mode of HIV infection**		
Sex worker	72 (54.5)	45.6-˗62,2
Others	60 (45.5)	36.8-˗54.3
**HIV status of the spouse**		
Positive	78 (65.0)	56.3-˗73.6
Negative	42 (35.0)	26.3-˗43.6
**Sexual contact with (n=100)**		
Spouse	84 (84.0)	54.8-˗71.8
Others	16 (16.0)	7.1-˗18.9
**Condom used during sex last 3 months (n=100)**		
Yes	74 (74.0)	65.2-˗82.7
No	26 (26.0)	17.2˗-34.7
**Ever forget to take ART medication**		
Yes	55 (41.7)	33.1-˗50.6
No	77 (58.3)	49.4-˗66.8

Of the total HIV infected people (PLHIV), onefourth (26.5%, 95% CI:18.9-34.1) were current smokers and one-fifth (22.7%, 95% CI: 15.5-30.0) were current alcohol drinkers (Table 1). The majority of participants (56.8%, 95% CI: 47.9-65.4) were diagnosed with HIV infection at an age ≤ 34 years; while 54.5% (95% CI: 45.6-63.2) of participants were found taking ART for less than a year after being diagnosed HIV positive (table not shown). Similarly, the majority of participants 54.5% (95% CI: 45.6-63.2) reported that they were infected from sex workers; 65.0% (95% CI: 56.3-73.6) of the participants’ spouses were HIV infected; 75.8% (95% CI: 68.3-83.2) of the participants were sexually active; 16.0% (95% CI: 7.1-18.9) had sexual contact with someone other than the spouse; and one-fourth of the participants (26.0%, 95% CI: 17.2-34.7) had sexual intercourse without using a condom (Table 1).

The morbidity of tuberculosis among HIV infected participants was 13.6% (95% CI: 7.7-19.6). Around half of the participants (52.3%, 95% CI: 43.4-61.0) were diagnosed as less than or equal to second clinical stage and the average (SD) CD4+T-cell count was 300.6 (SD=158.3) (table not shown). Two-fifths of the respondents (41.7%, 95% CI: 33.1-50.6) had ever forgotten to take ART medication (Table 1).

Table 2 shows significant differences between tobacco smoking and alcohol drinking with demographic, behavioral and health characteristics. Tobacco smoking was significantly higher among HIV infected males. Tobacco smoking was significantly different by occupation. Tobacco smoking was significantly different between alcohol drinkers and non-drinkers. The highest proportion of tobacco smoking was found among the PLHIV who were infected after sexual contact with sex workers, whereas a lower proportion was found amongst the respondents who had a HIV positive spouse; but a slightly higher proportion was found among those who had sexual contact with their infected spouse.

**Table 2:** Estimated prevalence by demographic-behavioral and health characteristics and substance use (n=132)

*Characteristics*	*Smoking (n=35) Yes (%)*	*Smoking (n=97) No (%)*	*p*	*Alcohol (n=30) Yes (%)*	*Alcohol (n=102) No (%)*	*p*
**Age in years**			0.84			0.84
≤ 36	21 (60.0)	56 (57.7)		17 (56.7)	60 (58.8)	
>36	14 (40.0)	41 (42.3)		13 (43.3)	42 (41.2)	
**Gender**			<0.01			1.00
Male	30 (85.7)	32 (33.0)		14 (46.7)	48 (47.1)	
Female	5 (14.3)	65 (67.0)		16 (53.3)	54 (52.9)	
**Ethnicity**			0.69			0.30
Non-indigenous	21 (60.0)	53 (54.6)		14 (46.7)	60 (58.8)	
Indigenous	14 (40.0)	44 (45.4)		16 (53.3)	42 (41.2)	
**Religion**			0.52			0.01
Hindu	23 (65.7)	70 (72.2)		15 (50.0)	78 (76.5)	
Others	12 (34.3)	27 (27.8)		15 (50.0)	24 (23.5)	
**Occupation**			0.03			0.43
Unemployed	6 (17.1)	37 (38.1)		8 (26.7)	35 (34.3)	
Informal employee	13 (37.1)	35 (36.1)		14 (46.7)	34 (33.3)	
Formal employee	16 (45.7)	25 (25.8)		8 (26.7)	33 (32.4)	
**Current tobacco smoking**						<0.01
Yes				14 (46.7)	21 (20.6)	
No				16 (53.3)	81 (79.4)	
**Current alcohol drinking**			<0.01			
Yes	14 (40.0)	16 (16.5)				
No	21 (60.0)	81 (83.5)				
**Duration of ART medication after diagnosis**		0.24			0.04	
< 1 Year	16 (45.7)	56 (57.7)		11 (36.7)	61 (59.8)	
≥ 1 Year	19 (54.3)	41 (42.3)		19 (63.3)	41 (40.2)	
**Mode of HIV infection**			<0.01			<0.01
Sex worker	32 (91.4)	40 (41.2)		24 (80.0)	48 (47.1)	
Others	3 (8.6)	57 (58.8)		6 (20.0)	54 (52.9)	
**HIV status of the spouse (n=120)**			<0.01			0.12
Positive	8 (26.7)	70 (77.8)		15 (51.7)	63 (69.2)	
Negative	22 (73.3)	20 (22.2)		14 (48.3)	28 (30.8)	
**Sexual contact with (n=100)**			<0.01			<0.01
Spouse	22 (70.9)	62 (89.9)		16 (61.6)	68 (91.9)	
Others	9 (29.1)	7 (10.1)		10 (38.4)	6 (8.1)	
**Condom used during sex last 3 months (n=100)**			0.46			<0.01
Yes	25 (80.6)	49 (71.0)		10 (38.5)	64 (86.5)	
No	6 (19.4)	20 (29.0)		16 (61.5)	10 (13.5)	
**Ever forget to take ART medication**			0.23			0.01
Yes	18 (51.4)	37 (38.1)		19 (63.3)	36 (35.3)	
No	17 (48.6)	60 (61.9)		11 (36.7)	66 (64.7)	

Alcohol use was significantly different by religion, tobacco smoking, and duration of ART medication. The highest prevalence of alcohol drinking was found among the respondents who were infected after sexual contact with sex workers; slightly higher prevalence was among those who had sexual contact with their spouse; and lower prevalence was among those who had sexual contact using condoms. Statistically significant difference was found with those variables. Alcohol drinking was higher among HIV infected individuals who ever forgot to take ART medication (Table 2).

Table 3 shows logistic regression results for tobacco smoking and alcohol drinking. Male participants were more likely to smoke tobacco (OR=12.19, 95% CI: 4.32-34.38, p<0.001) than females, and participants with formal employment were more likely (OR=3.95, 95% CI: 1.36-11.47, p=0.012) to smoke tobacco than those who were unemployed. The participants who were infected after sexual contact with sex workers were more likely (OR=15.2, 95% CI: 4.35-53.08, p<0.001) to smoke tobacco than those who were infected by other than sexual contact. The participants who lived with a HIV infected spouse were less likely (OR=0.10, 95% CI: 0.04-0.27, p<0.001) to smoke tobacco than those who lived with a HIV negative spouse.

**Table 3:** Logistic regression for substance use among HIV infected people (n=132)

*Characteristics*	*Tobacco smoking*			*Alcohol drinking*			
	*OR*	*95% CI*	*p*	*OR*	*95% CI*	*p*
**Age in years**						
≤ 36 – Ref.						
>36	0.91	0.41˗-2.0	0.815	1.09	0.48˗-2.49	0.833
**Gender**						
Male	12.19	4.32˗-34.38	<0.001	0.98	0.44˗-2.23	0.970
Female – Ref.						
**Ethnicity**						
Non-indigenous	1.25	0.57˗-2.73	0.583	0.61	0.27˗-1.39	0.240
Indigenous – Ref.						
**Religion**						
Hindu	0.74	0.32-˗1.69	0.477	0.31	0.13˗-0.72	0.007
Others – Ref.						
**Occupation**						
Unemployed – Ref.						
Informal employee	2.29	0.78-˗6.69	0.130	1.80	0.67-˗4.84	0.243
Formal employee	3.95	1.36˗-11.47	0.012	1.06	0.36˗-3.15	0.916
**Education**						
No formal education – Ref.						
Formal education	1.61	0.70˗-3.73	0.266	1.44	0.60-˗3.47	0.411
**Marital status**						
Single – Ref.						
Married	1.54	0.60-˗3.95	0.366	1.51	0.56-˗4.09	0.414
**Children**						
Yes	1.53	0.47-˗4.94	0.476	3.00	0.65˗-13.75	0.157
No – Ref.						
**Number of children**						
≤ 2 – Ref.						
>2	0.33	0.09-˗1.19	0.090	0.33	0.09˗-1.19	0.09
**Per capita income**						
≤ 5000 NPR – Ref.						
> 5000 NPR	1.09	0.49˗-2.44	0.825	2.19	0.95-˗5.01	0.064
**Current tobacco smoking**						
Yes	-	-	-	3.37	1.42˗-8.00	0.006
No – Ref.						
**Current alcohol drinking**						
Yes	3.37	1.42˗-8.00	0.006	-	-	-
No – Ref.						
**Age at HIV diagnosis**						
≤ 34 Years – Ref.						
> 34 Years	0.84	0.38˗-1.84	0.658	1.20	0.53-˗2.72	0.661
**Age at ART started**						
≤ 35 Years – Ref.						
> 35 Years	0.94	0.43-˗2.05	0.88	1.15	0.51-˗2.61	0.732
**Duration of ART medication**						
< 1 Years – Ref.						
≥ 1 Years	1.62	0.75˗-3.53	0.223	2.57	1.11-˗5.96	0.028
**Mode of HIV infection**						
Sex worker	15.2	4.35˗-53.08	<0.001	4.50	1.70˗-11.93	0.003
Others – Ref.						
**HIV status of spouse**						
Positive	0.10	0.04˗-0.27	<0.001	0.48	0.20-˗1.12	0.088
Negative – Ref.						
**Have sexual contact last 3 month**						
Yes	3.14	1.02˗-9.74	0.047	2.46	0.79˗-7.68	0.121
No – Ref.						
**Condom used during sex last**						
Yes	1.70	0.61-˗4.77	0.313	0.10	0.03-˗0.27	<0.001
No – Ref.						
**Clinical stages**						
≤ 2 Stage – Ref.						
>2 Stage	0.77	0.35˗-1.67	0.502	0.46	0.20˗-1.08	0.076
**Known morbidities**						
Tuberculosis	0.51	0.14˗-1.89	0.315	0.38	0.08-˗1.77	0.220
Others – Ref.						
**CD4+T-cell count**						
≤ 300 count – Ref.						
> 300 count	0.53	0.24˗-1.18	0.120	0.64	0.28-˗1.47	0.292
**Ever forget to take ART medication**						
Yes	1.72	0.79˗-3.74	0.174	3.17	1.36-˗7.38	0.008
No – Ref.						

OR = odds ratio, Ref. = reference, CI = confidence interval

Participants who followed the Hindu religion were less likely (OR=0.31, 95% CI: 0.13-0.72, p=0.007) to drink alcohol compared with those who followed another religion than Hinduism. Participants who were taking ART medication for more than or equal to a year were more likely (OR=2.57, 95% CI: 1.11-5.96, p=0.028) to drink alcohol than those who were taking medication for less than a year. Participants who were infected after sexual contact with sex workers were more likely (OR=4.50, 95% CI: 1.70-11.93, p=0.003) to drink alcohol than those who were infected by other than sexual contract. HIV infected individuals who used a condom during sexual contact were less likely (OR=0.10, 95% CI: 0.03-0.27, p<0.001) to drink alcohol. HIV infected individuals who ever forgot to take ART medication were three times more likely (OR=3.17, 95% CI: 1.36-7.38, p=0.008) to drink alcohol than those who never forgot to take ART medication (Table 3).

## DISCUSSION

The prevalence of tobacco smoking and alcohol drinking among HIV infected people was found to be higher than the general population. Our study revealed that the proportion of tobacco smoking (26.5%) and alcohol drinking (22.7%) among HIV infected people is high.

Male participants revealed the significant association with current tobacco smoking, followed by formal employment, and mode of HIV infection after having sexual contact with sex workers. Our study revealed a higher proportion of tobacco smoking among male PLHIV compared to the national rate^20^. One of the possible reasons of the low prevalence among female respondents could be under reporting in the data by females, which might not replicate the exact prevalence. The degree of under reporting of smoking status by females is unknown in low income countries with strong social and cultural apprehensions against tobacco smoking by females. Our findings revealed current smoking was associated with formal employment, contrary to previous findings that revealed that current smoking was associated with unemployment^21^. Most of those with formal employment can purchase tobacco more easily, which could be a possible reason, while HIV status might result in a weak economic situation resulting in a lack of money to purchase tobacco, as would also be among the unemployed. However, the result was a bit contradictory as the tobacco smoking prevalence was higher among the low-income group, though not statistically significant. Similarly, previous studies indicated that lower socioeconomic groups were more likely to smoke tobacco^21^. Current tobacco smoking had significantly high prevalence among those who were HIV infected from sex workers. Psychological and depressive states might be the possible reason for being more likely to smoke among PLHIV^22^. Tobacco smoking was low among PLHIV when both members of the couple were HIV infected. If the couple had a similar problem, they might have adjusted themselves to circumstances to avoid depression.

Alcohol drinking was more likely to be associated with current tobacco smoking among PLHIV. This finding indicated that either smoking or alcohol drinking encouraged an individual to trigger co-addiction, among HIV infected individuals. Previous literature revealed that tobacco smoking rate among HIV infected people was higher when someone smoked along with alcohol consumption^13,23,24^.

The longer the duration of ART medication after HIV diagnosis the more likely the drinking of alcohol, which might be due to more side effects at the beginning of ART medication. The feeling of uncertainty to be cured after ART medication and mental distress might be other reasons to indulge in alcohol drinking^25^. Our findings revealed that prevalence of alcohol drinking was significantly higher among the respondents who were HIV infected from sex workers. Psychological problems could be the main reason for drinking alcohol and persist after feeling of regret^25^. Previous studies have revealed a relationship between alcohol drinking and risky sexual behavior^19,26^. Another study revealed that sexual intercourse without a condom was associated with a higher likelihood for alcohol consumption among HIV infected people^27^. Our results are in agreement with these findings. Risky sexual behavior increased vulnerability to HIV transmission as well as to other infections. ART medication interruptions were related to the possibility of treatment failure^28^. Our study findings revealed non-adherence of ART medication was associated with alcohol drinking. Stopping ART medication might be because of the fear of interactive toxicity with alcohol that one felt after drinking^16^. Previous literature highlighted that non-adherence to ART, decline of CD4 cells count, and delayed on care of HIV were associated with alcohol consumption^17,18,29^.

This study has several strengths and limitations. We used probability sampling of HIV infected individuals; and assessed the self-reported information related to substance use and ART medication adherence. Due to the small sample size, we could not include different controlling variables in the regression model. Our findings were based on cross-sectional data and small sample size that limited us from drawing causal inferences. Obtaining information about sensitive behaviors like drug use through the face-to-face interview process could lead to underreporting as a result of social desirability biases. We did not collect initiation time of substance use and the quantity of the substance used. We did not use standardized tools to assess tobacco smoking, alcohol drinking and ART adherence. We did not measure the frequency of alcohol drinking and tobacco smoking, therefore we were unable to perform further analysis. The sample was extracted from a central level ART clinic and the participants were selected from those clients who visited there from across the country to get the services. Therefore, the findings of this study could not be generalized to other HIV infected populations in other settings. However, this study could simplify a significant proportion of substance use among HIV infected people in Nepal to some extent.

## CONCLUSIONS

The proportion of tobacco smoking and alcohol drinking is high among HIV infected people compared to the general population in Nepal. Low ART medication adherence is associated with alcohol drinking. There is an urgent need to develop tobacco cessation and alcohol avoidance cost-effective interventions for HIV infected people. Integrated counseling, tobacco cessation and motivational programs are needed in ART clinics and community settings, which might be helpful to improve the ART medication adherence and help HIV infected people to live longer.
